# Infectious diseases in pregnancy in Sri Lanka: gaps in surveillance, diagnosis, and policy: a scoping review

**DOI:** 10.1186/s12879-026-12650-x

**Published:** 2026-03-31

**Authors:** Thilini Agampodi, Hwa Young Kim, Dilrukshi Menike, Digantha Aswaddumage, Madushika Sewwandi, Janith Warnasekara, Dinesha Jayasundara, Indika Senavirathna, Chamila Kappagoda, Raphaël M. Zellweger, Suneth Agampodi

**Affiliations:** 1 Epidemiology, Public Health, Impact Unit, International Vaccine Institute, Seoul, 08826 Republic of Korea; 2 Department of Community Medicine, Faculty of Medicine and Allied Sciences, Rajarata University of Sri Lanka, Saliyapura, 50008 Sri Lanka; 3 Department of Microbiology, Faculty of Medicine and Allied Sciences, Rajarata University of Sri Lanka, Saliyapura, 50008 Sri Lanka; 4 Department of Biochemistry, Faculty of Medicine and Allied Sciences, Rajarata University of Sri Lanka, Saliyapura, 50008 Sri Lanka; 5 Innovation, Initiatives & Enterprise Development Department, International Vaccine Institute, Seoul, 08826 Republic of Korea

**Keywords:** Infectious diseases, Pregnancy, Sri Lanka, Scoping review, Disease burden

## Abstract

**Background:**

Infectious diseases during pregnancy are often overlooked in the Global South. Country-level mapping of infectious diseases and their effects on pregnancy would provide possible intervention strategies for diagnosis, prevention, and management. This scoping review aims to map the evidence from 2000–2024 on pathogens, mortality, morbidity, and adverse outcomes caused by infectious diseases in pregnancy among pregnant women in Sri Lanka.

**Methods:**

We conducted a PRISMA-ScR-based systematic search on PubMed, Scopus, EBSCO, and the Web of Science using key terms related to infectious diseases, pregnancy, and Sri Lanka. We hand-searched 32 local-indexed journals, library catalogs, and grey literature on Ministry of Health websites. Publications in English from January 2000 to March 2024 were included for title/abstract and full-text screening. We performed a narrative synthesis in reporting infectious diseases in pregnancy.

**Results:**

We synthesized data from 117 records, including 70 scholarly sources and 47 grey literature records, to map infectious diseases impacting pregnant women over the past two decades. The analysis identified 33 pathogens associated with 26 infectious diseases, with 19 conditions linked to maternal deaths. Dengue was the most frequently reported (15 of 70 studies reporting outcomes). Respiratory viruses accounted for nearly half (104/214) of reported maternal fatalities, while malaria, hepatitis B, HIV, and hepatitis E were less frequently documented. Most evidence came from tertiary-care centres, with limited representation of rural areas. This review highlights a decline in dengue-related maternal mortality following the introduction of national management guidelines. However, bacterial infections such as leptospirosis and tuberculosis remain significant contributors to maternal mortality. We identify key gaps, including deficiencies in diagnostics and screening for maternal conditions; research disparities; limited data availability; the absence of pregnancy-specific infectious disease surveillance in Sri Lanka; and shortcomings in classifying infectious diseases in morbidity and mortality surveillance.

**Conclusion:**

The data on infectious diseases in pregnant women in Sri Lanka show significant gaps, with available data systematically underrepresenting rural areas. Addressing research gaps, enhancing disease surveillance, and strengthening policy implementation is essential for ending preventable maternal mortality.

**Supplementary Information:**

The online version contains supplementary material available at 10.1186/s12879-026-12650-x.

## Introduction

Infectious diseases are a significant cause of morbidity and mortality during pregnancy worldwide. Deaths due to infectious diseases during pregnancy are often classified as “indirect” maternal deaths. The World Health Organization’s (WHO) revised definition of maternal sepsis emphasizes infectious diseases in pregnancy, equating their priority with sepsis during childbirth, post-abortion, or postpartum [1, 2]. Global data indicates that 33.9% of infection-related maternal hospital admissions and 27.9% of infection-related severe maternal outcomes occur during the antenatal period [3]. Nevertheless, infectious diseases in pregnancy are poorly identified, managed, and reported in LMICs [3]. Early identification and reporting of maternal infections during pregnancy contributes to Ending Preventable Maternal Mortality (EPMM) and alleviating consequences [4].

Pregnancy is a period during which women are particularly vulnerable to infectious diseases due to immunological adaptations [5, 6]. It has been shown that pregnant women are more susceptible to specific infectious diseases such as Listeriosis [7], Tuberculosis [8], and Malaria [9]. Many adverse outcomes can follow infectious diseases in pregnancy, including preterm birth [10], maternal sepsis, distress, and death [3, 6, 11], as well as vertical and ascending transmission of infection to the fetus and the newborn, which can cause congenital anomalies, neonatal sepsis, and pregnancy losses [12]. Control and prevention of infectious diseases in pregnancy have generated exceptional gains in maternal and fetal well-being, exemplified through vaccination (tetanus, rubella, influenza) [13], clinical management in dengue [14], other preventive measures such as WASH interventions in cholera [15], and vector control in malaria [16]. However, given the emerging and reemerging nature of communicable diseases, strengthening evidence generation for preventing morbidity and mortality is essential and urgently required.

Sri Lanka is considered exemplary in achieving maternal and child health services and indicators over the past century [17]. The country has a low maternal mortality compared to LMICs [18]. Maternal deaths due to direct obstetric causes have been reduced over the past decades, while indirect causes are on the rise. Infectious diseases have been a leading cause of indirect maternal deaths in Sri Lanka in recent years. Observable gaps exist in the country’s evidence on infectious diseases in pregnancy, including surveillance and availability of data, research, and development of appropriate management. A systematic evidence synthesis on infectious diseases in pregnancy in the country is an impending need to address these gaps in services and research. We aimed to map and synthesize the evidence on infectious diseases in pregnancy in Sri Lanka, emphasizing disease burden and their impact on maternal and fetal health.

## Methods

We conducted this scoping review according to the updated Joanna Briggs Institute (JBI) guidelines and reported it according to the PRISMA-ScR [19, 20]. The protocol was registered in the Open Science Framework: 10.17605/OSF.IO/PT4XQ.

### Inclusion criteria

We included peer-reviewed scholarly sources (journal articles, academic thesis, conference proceedings) and grey literature published by the Ministry of Health and related agencies in Sri Lanka (reports, circulars, institutional newsletters, guidelines). Eligible documents were published in English and contained primary or secondary data on infectious diseases among pregnant women in Sri Lanka from January 2000 to March 2024. We excluded articles reporting estimated data on maternal morbidity and mortality. Even if the full-text articles were unavailable, we included all abstracts available as conference proceedings, as the data on the theme were limited.

### Information sources

A comprehensive electronic search was conducted in PubMed, Scopus, EBSCO, and Web of Science using key terms related to infection, communicable diseases, pregnancy, and Sri Lanka. To account for the limited representation of local publications in indexed journals, we manually searched 32 non-indexed local journals (Supplementary Table 1) available in the Sri Lanka Journals Online database (SLJOL) and the Postgraduate Institute of Medicine (PGIM) library catalog. These sources were included as many relevant studies are published outside indexed databases. Additionally, grey literature was identified through manual searches of key national health institution websites, including the Epidemiology Unit, Family Health Bureau (FHB), National Sexually Transmitted Diseases and AIDS Control Programme (NSACP), Anti-Malaria Campaign, Anti-Filariasis Campaign, Anti-Leprosy Campaign, and the National Programme for Tuberculosis Control and Chest Diseases (NPTCCD) under the Ministry of Health, Sri Lanka. Publications from these sources containing data on pregnancy were included in the review.

### Search strategy

The search was structured around three key themes: (1) infection and types of infections/diseases, (2) pregnancy, and (3) Sri Lanka (Supplementary Table 2). A hand-search of 30 out of 32 journals in SLJOL was conducted using the term ‘pregnancy.’ At the same time, all titles in the Sri Lanka Journal of Obstetrics and Gynaecology and the Sri Lanka Journal of Perinatal Medicine were manually screened for relevant studies. Additionally, casebooks and theses related to infection and pregnancy were reviewed from the PGIM library catalog. Reports published by the Ministry of Health on the study theme were identified and extracted from institutional websites.

### Screening

Initial title and abstract screening were followed by a full-text screening using Rayyan, a web-based software for systematic reviews [21]. Two investigators evaluated each article at both title/abstract and full-text screening and consulted a third investigator on the absence of consensus. Criteria for excluding full-text articles were documented to allow transparency of the process.

### Data extraction

We extracted data using a data extraction sheet (Supplementary Table 3) to gather information on the publications’ characteristics, morbidity and mortality indicators, diagnostics, maternal complications, pregnancy and neonatal outcomes, and outcomes.

### Data analysis

A narrative analysis was conducted to describe the publication characteristics, identify evidence gaps, and describe morbidity, mortality, and reported outcomes of infectious diseases in pregnancy.

## Results

**Fig. 1:**
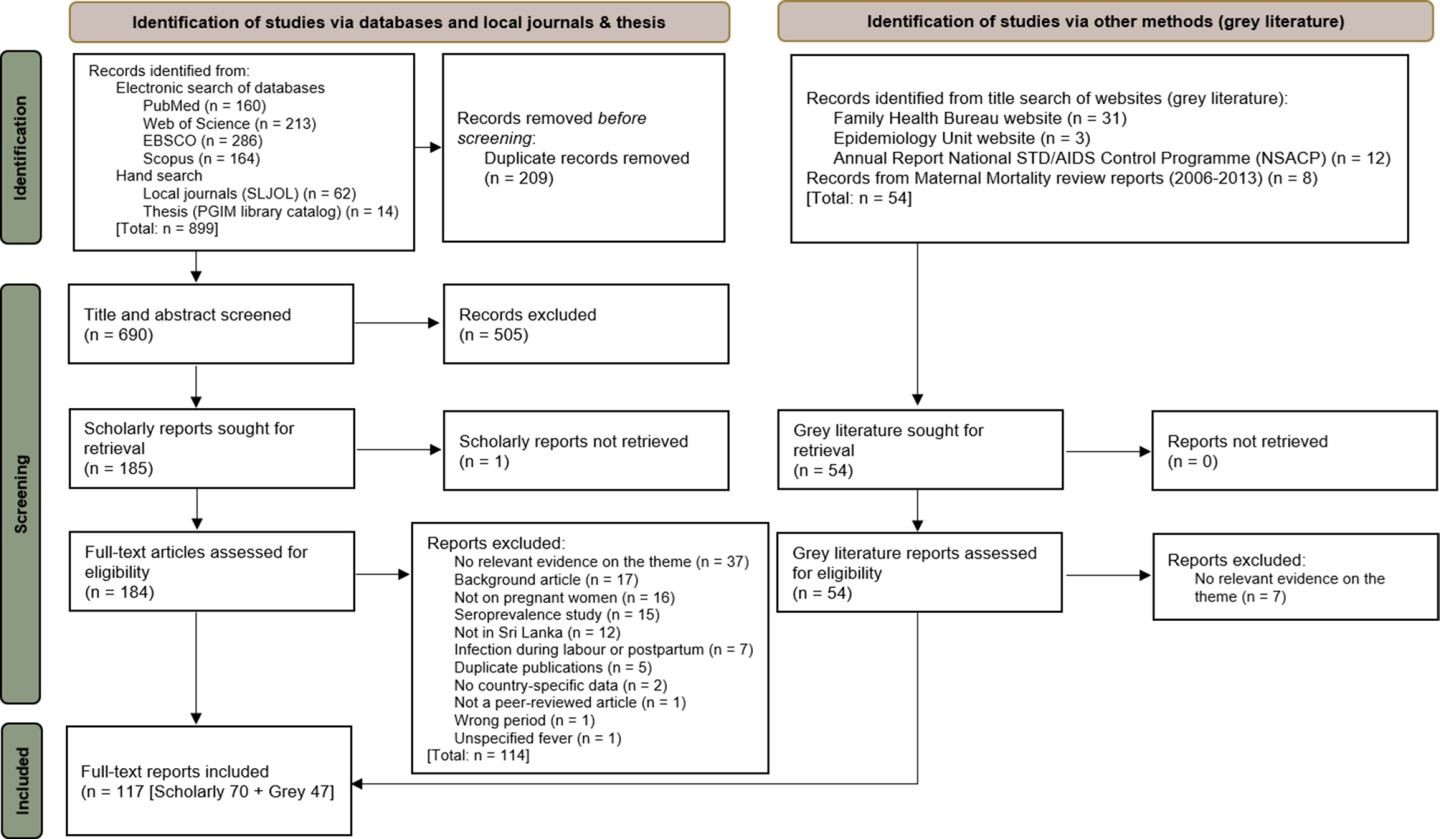
PRISMA flow diagram for the scoping review of infectious diseases in pregnancy in Sri Lanka, January 2000 – March 2024

We retrieved 953 records, including 899 from electronic database searches and 54 from grey literature searches. After removing 209 duplicates, 690 abstracts were screened, with 505 excluded. Full-text screening was performed on 238 records (184 from databases and 54 from grey literature). Of these, 121 were excluded (see Fig. 1 for details). A total of 117 records were included in the final synthesis.

### Composition and scope of reviewed scholarly and grey literature

The final dataset included 70 scholarly documents and 47 grey literature records. Of the 70 scholarly records, 53 (75.7%) were journal articles, while the remaining included conference abstracts (*n* = 8) [22–29], abstracts from postgraduate clinical case studies (*n* = 5) [30–34], theses (*n* = 4) [24, 35–37], and a book chapter [38]. Eight of the 70 scholarly documents reported national or institutional secondary data [38–45], while 61 reported primary data. Of the primary data articles, 63.9% (*n* = 39) were case reports or series, followed by 18 cross-sectional studies [23–26, 37, 46–54], three cohort studies [55–57], and one study with a case-control design [37]. The journals where the 53 articles and eight abstracts published were distributed across various disciplines: General Medicine (*n* = 17) [43, 44, 53, 58–70], Infectious Diseases/Tropical Medicine (*n* = 14) [23, 47, 50–52, 56, 59, 66, 71–76], Obstetrics and Gynecology (*n* = 11) [22, 25–28, 45, 46, 48, 77–79], Community Medicine/Public Health (*n* = 5) [38, 42, 49, 54, 57], Medico-legal (*n* = 3) [80–82], STI/Venerology (*n* = 4) [41, 83–85], Pediatrics (*n *= 3) [86–88], Anesthesiology (*n* = 1) [89], Perinatal (*n* = 1) [39], Respiratory Medicine (*n* = 1) [90], and Neurology (*n* = 1) [29]. Twenty-eight (53.8%) journal articles included were published in 13 local journals.

The grey literature records included 42 reports, three newsletters, one circular, and one guideline with case numbers. These records were produced by the Family Health Bureau (*n* = 33), Epidemiology Unit (*n* = 2), and the NSACP (*n* = 12) of the Ministry of Health, Sri Lanka. The reports included the Annual Reports of the Family Health Bureau 2010–2021 (*n* = 12), National Maternal Mortality Review reports 2006–2013 (*n* = 8), reports on Outcomes of Maternal Death Surveillance Response (MDSR) (*n* = 5), other reports related to maternal near misses, MDSR dissemination, and COVID-19 Pandemic produced by the FHB (*n* = 4), a Weekly Epidemiological Report published by the Epidemiology Unit, and Annual Reports of the NSACP (*n* = 12).

### Trends in journal publication outputs, focused diseases/conditions, and geographical coverage

The overall number of articles published remained relatively stagnant until 2015, with only a modest increase in dengue-related publications from 2010 to 2014. After 2015, there was an apparent rise in the total number of publications, even when excluding contributions from dengue and COVID-19. The evident high output after 2019 is primarily due to COVID-19-related publications.

**Fig. 2:**
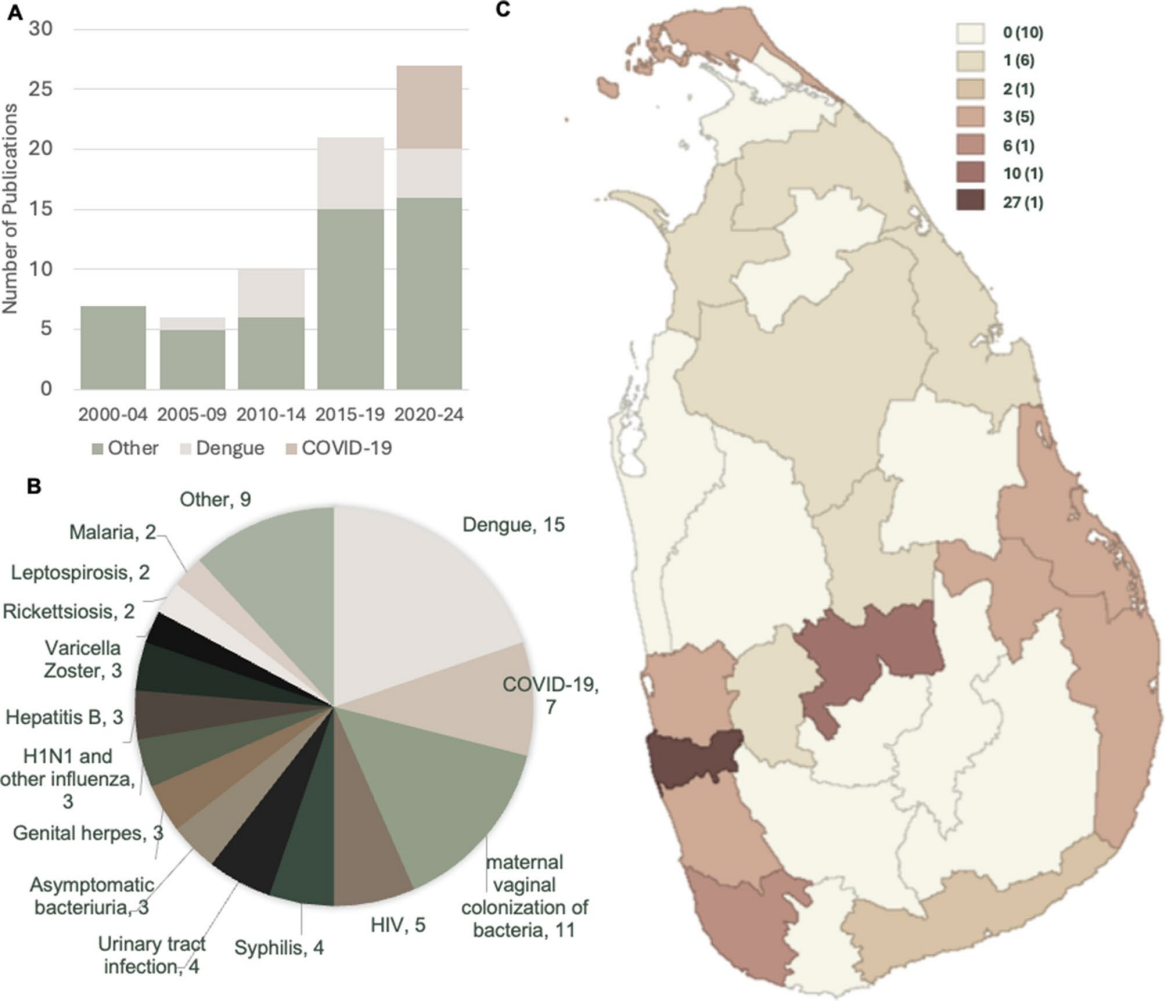
Distribution of infectious conditions in pregnancy investigated in published literature in Sri Lanka (2000–2024). A. Number of publications by year. B number of publications by disease/condition. C. Representation of different districts in published literature (other conditions in the 2B included chikungunya, filarial infection, unspecified infective hepatitis, Trichomonas vaginalis infection, tuberculosis, hepatitis C, viral myocarditis, and pelvic inflammatory disease)

Altogether, 26 different infectious conditions were reported in the published literature. The most studied topics were dengue (15 publications) [27, 28, 44, 62–64, 66–68, 71–73, 78, 86, 91] and maternal vaginal colonization of bacteria (11 publications) [23, 24, 37, 50, 51, 53, 55, 56, 70, 76, 88], followed by COVID-19 (7 publications) [26, 29, 39, 40, 61, 77, 87], HIV (5 publications) [32, 38, 41, 42, 84], syphilis [32, 38, 41, 85], urinary tract infections (in 4 publications each) [25, 35, 54, 57], and genital herpes [30, 31, 79], hepatitis B [22, 36, 74], H1N1 and other influenza [54, 58, 89], varicella-zoster infection [43, 54, 81], and asymptomatic bacteriuria (in 3 publications each) [47–49]. Less commonly reported were leptospirosis [65, 80], rickettsial diseases [59, 90], and malaria (in 2 publications each) [43, 75]. Diseases/conditions, chikungunya [69], filarial infection [52], unspecified infective hepatitis [46], Trichomonas vaginalis infection [34], tuberculosis [60], hepatitis C [83], viral myocarditis [45], and pelvic inflammatory disease were reported in one publication each (Fig. 2) [33]. Thirty-three pathogens were isolated, cultured, or confirmed related to these conditions. In three case reports on tuberculosis, varicella-zoster viral sepsis, and infective endocarditis, the diagnoses were made based on postmortem findings. The case reports on leptospirosis in pregnancy indicated that serological diagnosis by MAT was available only postmortem (Supplementary Table 5). While a few studies examined antibiotic sensitivity [23, 24, 47, 48, 51, 76], only one specifically investigated antimicrobial resistance (AMR) [56]. This study reported the isolation of four extended-spectrum beta-lactamase (ESBL)-producing coliforms from cases of asymptomatic vaginal colonization, which showed resistance to third- and fourth-generation cephalosporins.

In scientific publications utilizing primary data (*n* = 61), the study populations were drawn from 15 of Sri Lanka’s 25 districts. Colombo accounted for the largest proportion (44.2%, *n* = 27), followed by Kandy (18%, *n* = 11) and Galle (9.8%, *n* = 6). Ampara, Batticaloa, Gampaha, Jaffna, and Kalutara districts contributed three publications each.

### Prevalence of infectious diseases in pregnancy

For the evidence synthesis of the prevalence and incidence of infectious diseases during pregnancy, we included only reports or articles with confirmed disease diagnoses; self-reported or unconfirmed morbidities were excluded (Table 1). Only the latest prevalence values are included in the table for diseases with annual national data and reports. The methodological quality of studies was evaluated using the tool by Loney et al. [92] and categorized as high, moderate, or low quality for reporting prevalence. Of the primary data studies, only those by Vidanagama D and Simetca et al. [36, 43] had a sample size exceeding 250. Similarly, adequate representation of the reference population was observed only in these two studies and the study by Weerasingha et al. [52]. In contrast, all other studies were conducted in tertiary-care institutions.

**Table 1:** Prevalence of specific infectious diseases among pregnant women

Disease/condition	Study ID	Year of conduct	Participants description	Study setting	Case count		Sample	Prevalence		Quality***
**National estimates**										
HIV	NSACP	2023	Women registered in national maternal care programme (MCP)	Sri Lanka	23		226,446	0.01%		High
Syphilis	NSACP	2023	Women registered in MCP	Sri Lanka	123		198,246	0.06%		High
COVID-19	Kasthuriaarachchi K 2021 [39]	2020	Women registered in MCP	Sri Lanka	115		328,554**	0.04%		Moderate
		2021	Women registered in MCP	Sri Lanka	9,063		301,786**	3.0%		Moderate
Hepatitis B (surface antigen)	Epidemiology Unit 2023	2022	Women attending ANC	Sri Lanka	0		1,269	0.00%		High
**Local estimates**										
**Bact. Vaginosis**										
Unspecified	Weerasingha G 2001 [37]	2001	Women > 14 weeks of gestation	Maternal care institutions within Colombo	105*		342	30.7%		Moderate
	Galappaththi et al. 2018 [24]	2016–17	Women admitted to Obstetric (Obs.) ward (with PROM)	Teaching Hospital, Galle	131		144	91.0%		High
*Group B Streptococcus*	Dilrukshi et al. 2023 [70]	2019	Women attending ANC (POA > 35 weeks)	Four teaching hospitals in Colombo	45		175	25.7%		Moderate
	Dissanayaka et al. 2015 [51]	2011	Women attending ANC (POA 35–37 weeks)	Teaching Hospital Peradeniya	30		100	30.0%		Moderate
	Sapugahawattha et al. 2022 [50]	2021	Women admitted to ANC/Obs. ward	Teaching Hospital Kandy	28		250	11.2%		Moderate
	Mirshath MHM 2023 [23]	2021–22	Women admitted to Obs. ward (with PROM)	Teaching Hospital Batticaloa	27		216	12.5%		Moderate
	Galappaththi et al. 2018 [24]	2016–17	Women admitted to Obs. ward (with PROM)	Teaching Hospital, Galle	20*		144	13.9%		High
*Coliforms* (Unspecified)	Mirshath MHM 2023 [23]	2021–22	Women admitted to Obs. ward (with PROM)	Teaching Hospital Batticaloa	23		216	10.6%		Moderate
	Galappaththi et al. 2018 [24]	2016–17	Women admitted to Obs. ward (with PROM)	Teaching Hospital, Galle	21*		144	14.6%		
E-coli	Nanayakkara D 2018 [56]	2015–16	Women admitted to Obs. ward (for delivery)	Teaching Hospital, Peradeniya	14		250	5.6%		
*Klebsiella spp.*					31		250	12.4%		
*Gardnerella spp.*	Fernando et al. 2001 [55]	2001	Women attending ANC at < 25 POA and 32 POA	Teaching Hospital, Galle	2*	1*	196	1.0%	0.5%	
Diptheriods					70*	74*	196	35.7%	37.8%	
Lactobacili					182*	193*	196	92.9%	98.5%	
Aerobic Gram negative Bacilli					17*	61*	196	8.7%	31.1%	
Candida Sp.	Fernando et al. 2001 [55]	2001	Women attending ANC at < 25 POA and 32 POA	Teaching Hospital, Galle	29*	57*	196	14.8%	29.1%	
	Galappaththi et al. 2018 [24]	2016–17	Women admitted to Obs. ward (with PROM)	Teaching Hospital, Galle	14		144	9.7%		
Micrococci	Fernando et al. 2001 [55]	2001	Women attending ANC at < 25 POA and 32 POA	Teaching Hospital, Galle	10*	1*	196	5.1%	0.5%	
Coagulase negative staphylococci	Fernando et al. 2001 [55]	2001	Women attending ANC at < 25 POA and 32 POA	Teaching Hospital, Galle	24*	55*	196	12.3%	28.1%	
	Galappaththi et al. 2018 [24]	2016–17	Women admitted to Obs. ward (with PROM)	Teaching Hospital, Galle	54*		144	37.5%		
*Staphylococcus aureus*	Mirshath MHM 2023	2021–22	Women admitted to Obs. ward (with PROM)	Teaching Hospital, Batticaloa	10		216	4.6%		
	Galappaththi et al. 2018 [24]	2016–17	Women admitted to Obs. ward (with PROM)	Teaching Hospital, Galle	4*		144	2.8%		
**Asymptomatic Bacteriuria**										
Unspecified	Kodikara et al. 2009 [49]	2006	Women attending ANC (POA < 20 weeks)	De Soysa Maternity Hospital Colombo	8		205	3.9%		High
	Gurupuran et al. 2013 [25] (abstract)	2012	Women attending ANC	Teaching Hospital, Jaffna	10*		196	5.1%		Low
Coliform	Perera et al. 2012 [47]	2011	Women attending ANC (booking visit)	De Soysa Maternity Hospital, Colombo	6		250	2.4%		
*Staphylococcus aureus*					2		250	0.8%		
*Staph saprophyticus*					1		250	0.4%		
**Other**										
Hepatitis B (surface antigen)	Vidanagama D 2004 [36]	2002	Women attending ANC	Field ANCs of Colombo, Gampaha, Kalutara	0		1,855	0.0%		High
Malaria	Simetca et al. 2002 [43]	2000–01	Women admitted to Obs. ward	Mallavi hospital, Mullativu	137		677	20.2%		High
Varicella-zoster					1		677	0.2%		Moderate
Pyelonephritis					1		677	0.2%		Moderate
UTI	Gurupuran et al. 2013 (abstract) [25]	2012	Women attending ANC	Teaching Hospital Jaffna	24*		196	12.3%		Low

*Calculated case counts are included, as the article only mentioned the prevalence

**Denominator was from national data: total number of pregnant women registered with the maternal care program

***According to the tool by Loney et al. [92]

National data from 2023 reported HIV and syphilis prevalence rates of 0.01% and 0.06%, respectively [93]. While two cases of a hepatitis B-positive pregnant woman were documented [22, 74], neither a national study or a regional study involving 1,855 pregnant women from three districts in the Western Province detected hepatitis B surface antigen [36, 94]. In 2021, national data recorded 9,063 COVID-19 cases among pregnant women [95].

### Maternal deaths due to infectious diseases in Sri Lanka

**Fig. 3:**
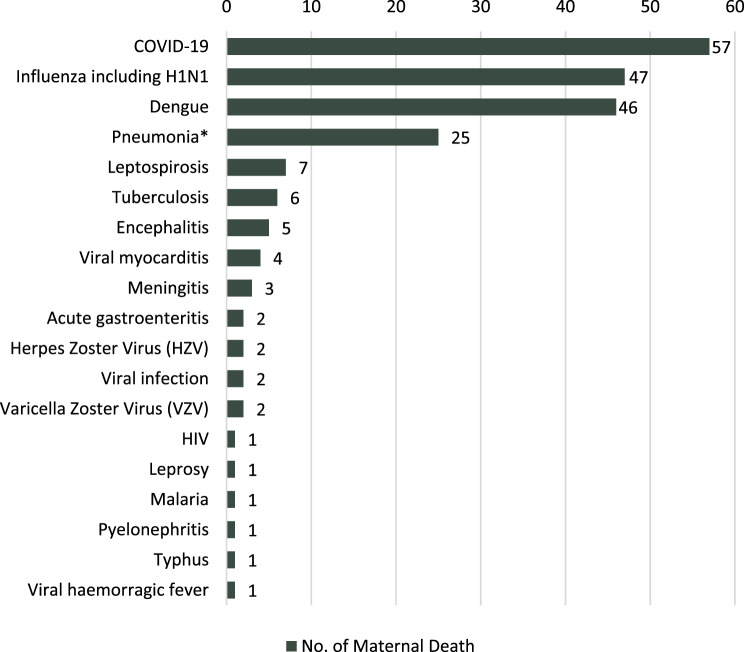
Infectious diseases contributing to maternal deaths (2006–2021). * Including bacterial and other unspecified viral pneumonia. (Maternal death counts were from different sources, and these figures do not represent verified national deaths attributed to each disease.)

National maternal death reporting lacked specificity regarding infectious causes. We compiled clearly defined maternal deaths reported in different sources due to infections, excluding unspecified sepsis, respiratory conditions, and other unspecified infectious causes (Fig. 3). The data presented here encompass maternal deaths among antenatal and postpartum women, not exclusively pregnant women, as these demarcations were not included in reports. From 2006 to 2021, we identified 214 reported maternal deaths attributed to infectious diseases (National Maternal Mortality Review reports 2006–2013, Annual Report of the Family Health Bureau 2010–2021, Outcomes of Maternal Death Surveillance and Response 2014–2017, other reports and circulars produced by the Family Health Bureau [see Supplementary Table 4]) [58, 65, 66, 80, 81, 86, 91]. The most frequently documented causes were COVID-19 (27%, *n* = 57), influenza (including H1N1) (22%, *n* = 47), dengue (22%, *n* = 46), pneumonia (12%, *n* = 25), and leptospirosis (3%, *n* = 7) (Fig. 3). Other infections linked to maternal deaths included acute gastroenteritis, herpes zoster, and chickenpox (each *n* = 2), as well as HIV, leprosy, malaria, pyelonephritis, and typhus (each *n* = 1).

### Maternal complications, pregnancy outcomes, and neonatal complications of infectious diseases in pregnancy

We compiled data on maternal complications, pregnancy outcomes, and neonatal outcomes associated with infectious diseases in pregnancy (Table 2). A total of 16 diseases/conditions had reports on at least one of these aspects, with dengue and COVID-19 being the only diseases documented across all three. Published reports primarily focused on maternal complications of dengue, which had the highest number and most diverse range of reported complications (*n* = 15). COVID-19 was the next most frequently reported (*n* = 6), followed by infective endocarditis (*n* = 5) and leptospirosis (*n* = 5). Severe maternal complications frequently highlighted in the literature included disseminated intravascular coagulation (DIC) in dengue, multiorgan failure and multisystem inflammatory syndrome in COVID-19, and pulmonary haemorrhage in leptospirosis, often linked to maternal deaths. Reports on adverse pregnancy outcomes most commonly addressed dengue, COVID-19, and chikungunya (*n* = 3 each). Intrauterine death (IUD), abortion, miscarriage, and preterm birth were recurrently mentioned across multiple diseases/conditions. Neonatal complications were frequently noted in dengue and COVID-19, particularly early neonatal deaths, while congenital anomalies were reported in syphilis, chikungunya, and COVID-19. Low birth weight, neonatal death, and respiratory distress were among the most commonly reported neonatal outcomes across various infectious diseases. Sri Lanka was identified as having eliminated mother-to-child transmission (MTCT) of HIV and syphilis by 2019 [38].

**Table 2:** Maternal complications, adverse pregnancy outcomes, and adverse neonatal outcomes reported among studies reporting maternal morbidities

Disease/condition	Maternal complications	Adverse pregnancy outcomes	Adverse neonatal outcomes	Study description
Dengue	Acute acalculous cholecystitis [78], Bleeding [66], DIC [86], Encephalopathy [62], Fetal distress [67], Haematuria [66], Hepatic encephalopathy [27], Intraperitoneal haemorrage [64], Intrauterine bleeding [68], Multiorgan failure [86, 91], Myocarditis [91], PROM [28], Pleural effusion [66], Unilateral sacroiliitis [63], Maternal death [66, 86, 91]	Abortion [26, 73, 91], IUD [86, 91], Stillbirth [27]	LBW [26], Neonatal death [68], Neonatal dengue [68, 71], Respiratory distress [67]	Cross-sectional 1 (*n* = 24) Case series 2 (*n* = 15, 26) Case reports 11
COVID-19	Bell’s palsy [29], Hyperpyrexia [61], Multiorgan failure [61], Multisystem inflammatory syndrome [77], Myocarditis [29], Pneumonia [26, 61]	Abortion [26, 95], Preterm labour [26, 87]	Facial nerve palsy [77], LBW [26], Multisystem inflammatory syndrome [77, 87]	Cross-sectional 1 (*n* = 69) Case reports 4
Genital herpes	Cervicitis [31], Genital ulcers [79], Recurrent episodes of HSV [79], Secondary bacterial infection [31]	Not mentioned	Congenital herpes [79], Neonatal death [79]	Case reports 3
HIV	Oral candidiasis, Weight loss [84]	Not mentioned		Case report 1
Infective endocarditis	Cardiogenic shock [82], Maternal death [82]	IUD [82]	Not mentioned	Case report 1
Leptospirosis	Focal hepatic necrosis [80], Maternal death [65, 80], Pulmonary haemorrhage [80], Respiratory distress [65], Coagulopathy [65]	Abortion [65]	Not mentioned	Case reports 2
Varicella zoster viral sepsis	Maternal death [81], Multiorgan failure [81], Septic shock [81]	IUD [81]	Not mentioned	Case report 1
Streptococcus pneumoniae infection	Chorioamnionitis [76], PROM [76]	Not mentioned	Birth asphyxia, Neonatal death [76],	Case series 1 (*n* = 2)
Chikungunya		Abortion, IUD, Preterm labour [69]	Congenital heart disease, Failure to thrive, Meningoencephalitis, Myocarditis, Pigmentation, Respiratory distress, Splenomegaly [69]	Follow-up study 1 (*n* = 50)
H1N1 influenza	Respiratory distress [89]	Not mentioned	Not mentioned	Case reports 2
Influenza A	Acute hemorrhagic pancreatitis [58], Maternal death [58]	Not mentioned	Not mentioned	Case report 1
Infective hepatitis	Maternal death [46]			Cross-sectional 1 (*n* = 8)
Rickettsial disease	Bilateral sensorineural deafness [59]	Not mentioned	Not mentioned	
Syphilis	Latent syphilis [32], Secondary syphilis [85]		Congenital syphilis [32, 41]	Case reports 3
Tuberculosis	Cardio respiratory arrest [60], Maternal death [60]	Not mentioned	Not mentioned	Case report 1
Vaginal colonization in PROM		Not mentioned	Neonatal sepsis [24], Neonatal colonization [56]	Cross-sectional 2 (*n* = 141, 250)
Vaginal colonization		Preterm labour [55]	LBW [55]	Cohort 1 (*n* = 201) Case-control 1 (*n* = 266 × 2)

### Susceptibility of pregnant women to selected infectious diseases

Though not included in the main study, we compiled data from 11 additional studies reporting the seroprevalence of infectious diseases among pregnant women (Table 3). In 97% of pregnant women, cytomegalovirus (CMV) antibodies were detected, indicating early exposure [96]. Rubella seroprevalence varied considerably, ranging from 31.6% to 82.0% in 2002–2003 and increasing to 88.1% in 2021 [97–100], suggesting reduced susceptibility over time. Hepatitis E showed a low seroprevalence (0.4%), indicating high susceptibility and low endemicity [101]. Similarly, there was high susceptibility for toxoplasmosis (70.1–87.8%) across regions [102, 103]. In contrast, measles, mumps, and varicella-zoster virus exhibited relatively high seroprevalence, with susceptibility ranging from 8.5% to 34% [99, 104, 105].

**Table 3:** Susceptibility of pregnant women to selected infectious diseases

Disease	Author ID	Participants	Study setting	Sero positives	Sample	Seroprevalence	Susceptibility**	Quality
Cytomegalovirus (CMV)	Abeynayake et al. 2017 [96]	Women attending ANC	De Soyza Maternity Hospital, Colombo	374	385	97.1%	2.9%	High
Rubella	Palihawadana 2002 [97]	Women attending ANC	Kaluthara District	149	471	31.6%	68.4%	High
	Weerasekera et al. 2003 [105]	Women attending ANC	Colombo South Teaching Hospital	410*	500	82.0%	18.0%	Moderate
	Palihawadana et. al 2003 [98]	Women attending ANC	Kaluthara District	471*	620	76.0%	24.0%	High
	Muthiah et al. 2021 [99]	Women admitted for delivery	De Soyza Maternity Hospital, Colombo and Castle Street Hospital for Women	259*	294	88.1%	11.9%	Moderate
Hepatitis E	Akram et al. 2021 [100]	Women attending ANC	Castle Street Hospital for Women	1	260	0.4%	99.6%	Low
Toxoplasmosis	Chandrasena et al. 2016 [101]	Women attending ANC	Colombo North Teaching Hospital	36	293	12.3%	87.8%	High
	Iddawela et al. 2017 [102]	Women attending ANC	Teaching Hospital Peradeniya	160	536	29.9%	70.1%	High
Measles	Muthiah et al. 2021 [99]	Women admitted for delivery	De Soyza Maternity Hospital, Colombo and Castle Street Hospital for Women	269*	294	91.5%	8.5%	Moderate
Mumps	Muthiah et al. 2021 [99]	Women admitted for delivery	De Soyza Maternity Hospital, Colombo and Castle Street Hospital for Women	261*	294	88.8%	11.2%	Moderate
Varicella-zoster virus	Premathilake et al. 2019 [103]	Women attending ANC	De Soyza Maternity Hospital, Colombo	254	385	66.0%	34.0%	High
	Daulagala et al. 2017 [104]	Women attending ANC	Teaching Hospital, Peradeniya	141	181	77.9%	22.1%	High

*The number of cases was not mentioned in the article

**Calculated susceptibility

## Discussion

This review synthesizes more than two decades of reported evidence on infectious diseases in pregnancy in Sri Lanka, mapping available data on morbidity, mortality, pathogens, maternal complications, and pregnancy outcomes. The reviewed literature identifies 33 pathogens linked to 26 infectious diseases, with 19 conditions associated with maternal deaths. Respiratory viruses accounted for nearly half (104/214) of reported maternal deaths, while malaria, hepatitis B, HIV, and hepatitis E were infrequently documented. High routine immunization coverage is reflected in the low reported susceptibility to measles and rubella, yet bacterial infections such as leptospirosis and tuberculosis remain documented contributors to maternal mortality. However, significant gaps and inequities in research are evident, with limited data on several infections and a lack of pregnancy-specific infectious disease surveillance. These findings highlight the need for strengthened research and systematic reporting to generate a more comprehensive understanding and support global maternal health initiatives, including pregnancy-specific vaccine development and policies for EPMM [106, 107].

The burden of infectious diseases in pregnancy is disproportionately high in LMICs [108] including South Asia, where conditions such as HIV [109], syphilis [110], hepatitis B [111], hepatitis E [112], malaria [113], dengue [114], tuberculosis [115], and Group B Streptococcus [116] significantly impact maternal and neonatal health. However, direct comparisons across countries remain challenging due to the lack of routine surveillance data on infectious diseases in pregnancy. Our review reflects this limitation in Sri Lanka, where the low endemicity of some infections and the country’s relatively low maternal mortality ratio (28.8/100,000 live births) [117] compared to surrounding countries in the region (102–175/100,000 live births) [118] may contribute to the limited number of available studies.

Sri Lanka has a longstanding infectious disease notification system under the Quarantine and Prevention of Diseases Ordinance (1897) and has achieved high immunization coverage through the Expanded Program on Immunization (EPI) [119], ensuring protection against vaccine-preventable infections like rubella and hepatitis B [120]. Additionally, the integration of infectious disease control within maternal health services has placed Sri Lanka at commendable standards compared to other LMICs [121]. For instance, while guidelines for dengue management in pregnancy were introduced in 2019 and adherence to them has reduced maternal mortality [64, 71, 72, 91, 122], other vector-borne diseases like chikungunya, which cause high rates of adverse pregnancy and neonatal outcomes [69], lack targeted policies. Similarly, Sri Lanka’s pandemic preparedness strategies have addressed maternal health to some extent, yet future preparedness efforts must incorporate broader maternal infection control measures to mitigate excess maternal deaths due to respiratory pathogens [106, 123].

Early diagnosis of infectious diseases is crucial in preventing adverse pregnancy and neonatal outcomes, yet the review highlights significant gaps in diagnostic access and early detection. Routine antenatal screening for HIV and syphilis has been rendered to detect mothers with asymptomatic infection and start on appropriate treatment, which has been beneficial to both mother and in the prevention of mother-to-child transmission (MTCT) [41]. However, in diseases such as leptospirosis and tuberculosis, which are still contributing to a significant number of maternal deaths in Sri Lanka, the few case reports indicate women presenting with severe disease identified only postmortem [60, 65, 80]. Comparing with the global scientific evidence, we also highlight the lack of guidelines for screening for important maternal conditions such as GBS colonization [124], detection of asymptomatic bacteriuria [125], and studies on AMR [126], as deficiencies in Sri Lanka.

Apart from the continuous disease surveillance data by NSACP, on HIV and syphilis, the data on the prevalence of morbidities are limited in the national program websites. Sri Lanka possesses a system for disease surveillance mediated by the Epidemiology Unit and separate vertical programs for Tuberculosis, Malaria, Leprosy, and Filariasis. However, none of these systems specified pregnant women. Maternal mortality review reports lack detailed cause-specific data, and classification inconsistencies hinder the accurate collation of infectious disease-related maternal deaths. For instance, grouping infectious respiratory diseases with non-infectious conditions like asthma, failing to differentiate antenatal sepsis from puerperal sepsis, and not identifying the underlying cause in pneumonia, sepsis, meningitis, and encephalopathy were some of these gaps (Annual Reports of the Family Health Bureau 2013–2021: Supplementary Table 4).

There is a significant geographical skew in research, with Colombo accounting for 44.2% of primary data studies, while ten districts had no documented research. This suggests that disease burden in underserved regions remains largely unknown, potentially leading to underestimation of risk and inadequate policy responses. The over-reliance on tertiary-care hospitals also limits generalizability, as primary-care settings—where access to diagnostics and specialist care is constrained—are underrepresented. Strengthening community-based surveillance and conducting multi-site studies are necessary to obtain a more comprehensive understanding of the true burden and distribution of infectious diseases in pregnancy across Sri Lanka.

This review has several limitations. First, the types, prevalence, and causative pathogens of infectious disease morbidities in pregnancy are derived solely from published studies, apart from HIV and syphilis, and may not fully represent the actual burden of infectious diseases among pregnant women in Sri Lanka. As noted, deficiencies in mortality classification systems also limit the accurate reporting of disease-specific maternal deaths. The geographical concentration of studies largely reflects the distribution of major academic institutions and tertiary hospitals and should not be interpreted as indicative of spatial patterns of disease occurrence. Although our search strategy was comprehensive and supplemented by a hand-search of local journals, some grey literature sources may not have been captured if they were unavailable through institutional or disease-specific websites. Further, the grey literature reports are although official Ministry of Health surveillance outputs, they are not peer-reviewed and reliance on them may introduce bias. However, we have minimized these biases through exclusion of estimated data and unverified morbidity reports. Overall, this review synthesises publicly accessible data and highlights substantial gaps and limited availability of reliable evidence on infectious diseases in pregnancy in Sri Lanka.

Addressing the gaps identified in this review requires an integrated approach combining maternal health programs with infectious disease control. Key priorities include strengthening surveillance to capture pregnancy-specific morbidity and mortality, expanding antenatal screening to include GBS, asymptomatic bacteriuria, and AMR-related infections, enhancing laboratory capacity for timely diagnosis and point-of-care testing, developing national guidelines for underreported infections such as leptospirosis and tuberculosis, improving research representation in rural districts, and integrating maternal infection preparedness into national pandemic response strategies.

## Conclusion

This review provides the first comprehensive synthesis of infectious diseases in pregnancy in Sri Lanka, highlighting significant gaps in research, surveillance, and policy implementation. The findings emphasize the urgent need for a proactive, evidence-based approach to maternal infection control to reduce morbidity and mortality. Strengthening disease surveillance, expanding screening, and improving diagnostic capacity are essential to bridging these gaps. Addressing these challenges will be critical for achieving global maternal health goals and ensuring effective infectious disease control at national and regional levels.

## Electronic supplementary material

Below is the link to the electronic supplementary material.

Supplementary Material 1

## Data Availability

The datasets used and/or analysed during the current study are available from the corresponding author on reasonable request.
