# Supplementary material for: Infectious diseases in pregnancy in Sri Lanka: gaps in surveillance, diagnosis, and policy: a scoping review
Source: BMC Infect Dis. 2026 Mar 31;26:681. doi: 10.1186/s12879-026-12650-x (PMC13041171; doi:10.1186/s12879-026-12650-x)
Supplement: Supplementary file 1 — Supplementary Material 1 [file 12879_2026_12650_MOESM1_ESM.docx]

**Supplementary Tables**

**Table of Contents**

[**Supplementary Table 1: The Sri Lanka Journals Online included in the search** 2](#_Toc201226296)

[**Supplementary Table 2: Search strategy, including the key terms** 3](#_Toc201226297)

[**Supplementary Table 3: Data extraction sheet variables** 4](#_Toc201226298)

[**Supplementary Table 4: Grey Literature included in the review** 5](#_Toc201226299)

[**Supplementary Table 5: Laboratory tests and procedures used for pathogen identification in infectious diseases in pregnancy** 6](#_Toc201226300)

**Supplementary Table 1: The Sri Lanka Journals Online included in the search**

| **Name of Journal** |
| --- |
| 1. Sri Lanka Journal of Obstetrics and Gynaecology |
| 1. Sri Lanka Journal of Perinatal Medicine |
| 1. University of Colombo Review |
| 1. Sri Lanka Journal of Urology |
| 1. Sri Lanka Journal of Venereology |
| 1. Sri Lanka Journal of Sexual Health and HIV Medicine |
| 1. Sri Lankan Journal of Anaesthesiology |
| 1. Sri Lanka Journal of Health Sciences |
| 1. Sri Lankan Journal of Infectious Diseases |
| 1. Sri Lankan Journal of Nursing |
| 1. Sri Lanka Journal of Health Research |
| 1. Sri Lanka Journal of Medicine |
| 1. Sri Lanka Journal of Neurology |
| 1. Medico-Legal Journal of Sri Lanka |
| 1. Sabaragamuwa University Journal |
| 1. Sri Lanka Journal of Critical Care |
| 1. Sri Lanka Journal of Dermatology |
| 1. Journal of the Ruhunu Clinical Society |
| 1. Journal of the Ceylon College of Physicians |
| 1. Journal of the College of Community Physicians of Sri Lanka |
| 1. Journal of the National Science Foundation of Sri Lanka |
| 1. Journal of the Postgraduate Institute of Medicine |
| 1. Journal of the University of Ruhuna |
| 1. Journal of Science of the University of Kelaniya |
| 1. KDU Journal of Multidisciplinary Studies |
| 1. Jaffna Medical Journal |
| 1. Journal of Diagnostic Pathology |
| 1. Anuradhapura Medical Journal |
| 1. Asian Journal of Internal Medicine |
| 1. Ceylon Journal of Medical Science |
| 1. Galle Medical Journal |
| 1. Ceylon Medical Journal |

**Supplementary Table 2: Search strategy, including the key terms**

| **Key terms** |
| --- |
| #1. Infection and types of infections and diseases  cholera OR "vibrio cholerae" OR polio OR "acute flaccid paralysis" OR measles OR rubella OR "German measles" OR chickenpox OR varicella OR shingles OR "herpes zoster" OR dengue OR diphtheria* OR corynebacterium OR typhoid OR "enteric fever" OR salmonella OR "abdominal typhus" OR paratyphoid OR encephalitis OR "Japanese encephalitis" OR rabies OR leptospirosis OR leptospira OR "Weil's disease" OR "weils disease" OR leishmania* OR "Kala- azar" OR leprosy OR "hansen's disease" OR malaria OR plasmodium OR meningitis OR mumps OR parotitis OR parotiditis OR tetanus OR tetani OR clostridium OR tuberculosis OR mycobacterium OR typhus OR Louse-Borne OR rickettsia OR "scrub typhus" OR hepatitis OR pertussis OR "whooping cough" OR bordetella OR pneumonia OR Bacteremia OR septicemia OR sepsis OR epiglottitis OR pericarditis OR myocarditis OR endocarditi* OR “human papilloma virus” OR influenza OR H1N1 OR flu OR "swine flu" OR COVID-19 OR "COVID 19" OR corona OR SARS-CoV-2 OR “SARS CoV 2” OR herpes OR "herpes simplex" OR HSV-1 OR HIV OR “human immuno deficiency” OR AIDS OR toxoplasmosis OR toxoplasma OR Zika OR CMV OR cytomegalo* OR syphilis OR chancre OR listeria OR listeriosis OR chlamydia OR chlamydiosis OR campylobacter OR candida OR candidiasis OR "white discharge" OR hookworm OR ancylostom* OR helminth OR "worm infestation" OR "rheumatic fever" OR streptococcus OR trichomoniasis OR trichomonas OR chikungunya OR filariasis OR elephantiasis OR filaria OR bacteriuria OR UTI OR "urinary tract infections" OR pyelonephriti* OR cystitis OR aspergillus OR gonorrhea OR klebsiella OR enterobacteriaceae OR fever OR pyrexia OR infection* OR viral OR virus OR bacterial OR fungal OR mycoses OR zoonoses OR parasitic OR “mosquito borne” OR "sexually transmitted diseases" OR STI  #2. Pregnancy  "maternal" OR "pregnancy" OR "pregnant" OR "obstetric" OR "antenatal" OR "ante natal" OR "perinatal" OR "peri natal" OR "gestation" OR "gestational" OR "peripartum" OR "peri partum" OR "prenatal" OR "pre natal"  #3. Sri Lanka  "Sri Lanka" OR Ceylon |
| **Search strategy** |
| #1 AND #2 AND #3 |

**Supplementary Table 3: Data extraction sheet variables**

| **Details of the publication**   - Title - Author - Institution (government/university/research institution) - Year of publication - Type of publication (journal article/report/textbooks/etc.) - Disease or condition reported - Discipline of medicine reporting/leading the research |
| --- |
| **Study characteristics (only for scientific studies)**   - Setting (clinic/community-based/hospital/multicenter/etc.) - Design (case report/case series/case-control/cohort/cross-sectional) - Participants (POG) |
| **Laboratory test(s) for identifying the disease** |
| **Epidemiological findings**   - Frequency (no. of cases/deaths) - Underlying population/sample - Proportion/percentage - Distribution (geographical area) |
| **Outcomes**   - Outcomes of individual diseases (pregnancy/neonatal/complications) [according to availability of studies reporting outcomes] |

**Supplementary Table 4: Grey Literature included in the review**

| **Serial** | **Title** | **Reported Year** | **Type** |
| --- | --- | --- | --- |
| **Family Health Bureau, Ministry of Health: URL -** [**https://fhb.health.gov.lk**](https://fhb.health.gov.lk) **[accessed March 2023]** | | | |
| 1 | Annual Report of FHB | 2010 | Report |
| 2 | Annual Report of FHB | 2011 | Report |
| 3 | Annual Report of FHB | 2012 | Report |
| 4 | Annual Report of FHB | 2013 | Report |
| 5 | Annual Report of FHB | 2014 | Report |
| 6 | Annual Report of FHB | 2015 | Report |
| 7 | Annual Report of FHB | 2016 | Report |
| 8 | Annual Report of FHB | 2017 | Report |
| 9 | Annual Report of FHB | 2018 | Report |
| 10 | Annual Report of FHB | 2019 | Report |
| 11 | Annual Report of FHB | 2020 | Report |
| 12 | Annual Report of FHB | 2021 | Report |
| 13 | Maternal Death Investigation reports | 2006 | Report |
| 14 | Maternal Death Investigation reports | 2007 | Report |
| 15 | Maternal Death Investigation reports | 2008 | Report |
| 16 | Maternal Death Investigation reports | 2009 | Report |
| 17 | Maternal Death Investigation reports | 2010 | Report |
| 18 | Maternal Death Investigation reports | 2011 | Report |
| 19 | Maternal Death Investigation reports | 2012 | Report |
| 20 | Maternal Death Investigation reports | 2013 | Report |
| 21 | Outcome of Maternal Death Surveillance and Response -2016 | 2016 | Report |
| 22 | Outcome of Maternal Death Surveillance and Response -2014 | 2014 | Report |
| 23 | Outcome of Maternal Death Surveillance and Response -2018 | 2018 | Report |
| 24 | Outcome of Maternal Death Surveillance and Response -2019 | 2019 | Report |
| 25 | Outcome of Maternal Death Surveillance and Response -2020 | 2020 | Report |
| 26 | MCH Quarterly October 2019 | 2018 | News letter |
| 27 | MCH Quarterly July 2017 | 2016 | News letter |
| 28 | MCH Quarterly April 2016 | 2015 | News letter |
| 29 | Findings of Maternal death reviews 2011-12 | 2011-12 | Report |
| 30 | National Maternal  Mortality  Reviews - 2016 | 2016 | Report |
| 31 | Prevention of maternal morbidity and mortality due to seasonal influenza | 2019 | Circular |
| 32 | Tracking of COVID- 19 positive pregnant women in Sri Lanka - Second Interim Report | 2022 | Report |
| 33 | Learning from the pregnant mothers just escaped from death- Maternal near misses in Sri Lanka | 2014 | Report |
| **Epidemiology Unit, Ministry of Health: URL -** [**https://www.epid.gov.lk**](https://www.epid.gov.lk) **[Accessed March 2023]** | | | |
| 34 | Flash back 2022_WER 31st-6th Jan 2023 | 2022 | Report |
| 35 | Guidelines for Clinical Management of Dengue Infection in Pregnancy | 2019 | Guideline |
| **National STI/AIDS Control Program: URL -** [**https://www.aidscontrol.gov.lk**](https://www.aidscontrol.gov.lk) **[Accessed March 2023]** | | | |
| 36 | Annual Report NSACP | 2012 | Report |
| 37 | Annual Report NSACP | 2013 | Report |
| 38 | Annual Report NSACP | 2014 | Report |
| 39 | Annual Report NSACP | 2015 | Report |
| 40 | Annual Report NSACP | 2016 | Report |
| 41 | Annual Report NSACP | 2017 | Report |
| 42 | Annual Report NSACP | 2018 | Report |
| 43 | Annual Report NSACP | 2019 | Report |
| 44 | Annual Report NSACP | 2020 | Report |
| 45 | Annual Report NSACP | 2021 | Report |
| 46 | Annual Report NSACP | 2022 | Report |
| 47 | Annual Report NSACP | 2023 | Report |

**Supplementary Table 5: Laboratory tests and procedures used for pathogen identification in infectious diseases in pregnancy**

| **Disease** | **Pathogen/s isolated or confirmed** | **Laboratory tests and procedures for diagnosis** |
| --- | --- | --- |
| Dengue | Dengue virus | Rapid antigen detection with NS-1 test, rapid detection of antibodies via lateral flow assays |
| Covid-19 | Severe Acute Respiratory Syndrome Coronavirus 2 (Sars-Cov-2) | Rapid antigen tests and PCR studies |
| Asymptomatic bacteriuria and UTI | *Staphylococcus* saprophyticus and other Staphylococci spp *Streptococcus* sp , *Enterococcu*s sp.and Coliform sp | Urine dipstick test and urine culture |
| Chikungunya | Chikungunya virus | Hemagglutination inhibition assay (HAI) in mothers and IgM levels in newborns |
| Filariasis | Filaria parasite | CFA detection by ICT cards, Eosinophilia of >10% |
| Genital herpes | Herpes simplex virus | Detection of giant cells by light microscopy |
| Influenza A | Influenza A subtype H3, subtype H1N1 | PCR studies followed by genotyping when indicated |
| Hepatitis B | Hepatitis B virus | Panel testing for hepatitis B with quantification of viral load by qPCR assays |
| HIV | Human Immuno Deficiency Virus | Serological assays and viral load confirmation with qPCR studies |
| Leptospirosis | *Leptospira* sp. | High serum Leptospira microscopic agglutination test (MAT) titers |
| Malaria | *Plasmodium vivax* | Microscopic examination of thin and thick blood films for malaria parasites |
| Vaginal colonization | Group B Streptococci, Anaerobic Gram negative bacilli, Candida sp., Coagulase negative S*taphylococcus* sp, Coliforms, Diptheriods, *E.Coli*, *Gardnerella* sp., *Klebsiella* spp., Lactobacilli, Micrococci, *Streptococcus pneumoniae* | Screening of vaginal swabs/ rectal swabs of mothers by culture on laboratory media and subsequent identification by either conventional laboratory methods or advanced techniques like MALDI-TOF-MS and PCR |
| Staphylococcal aureus sepsis | *Staphylococcus aureus* | Blood Culture and swab cultures from skin lesions |
| Hepatitis C | Hepatitis C virus | Serological confirmation, viral load assay by PCR to quantify the viremia |
| Rickettsial disease | *Rickettsia* Sp. | Weil-Felix test followed by confirmation with IFA |
| Syphilis | *Treponema pallidum Spp pallidum* | VDRL and TPPA assays |
| Trichomonas vaginalis infection | *Trichomonas vaginalis* | organism detection in wet vaginal smears and urine |
| Tuberculosis | *Mycobacterium tuberculosis* | Postmortem finding of granulomatous lung lesions with central caseation necrosis |
| Varicella zoster infection | Varicella zoster virus | Postmortem finding of vesicular skin lesions distributed along a dermatome with some diffuse blisters in other body areas |
